# Acute kidney injury in severe trauma assessed by RIFLE criteria: a common feature without implications on mortality?

**DOI:** 10.1186/1757-7241-18-1

**Published:** 2010-01-05

**Authors:** Ernestina Gomes, Rui Antunes, Cláudia Dias, Rui Araújo, Altamiro Costa-Pereira

**Affiliations:** 1 Unidade de Cuidados Intensivos Polivalente, Hospital de Santo António, Centro Hospitalar do Porto, 4099 - 001 Porto, Portugal; 2 Serviço de Bioestatística e Informática Médica, Faculdade de Medicina da Universidade do Porto, Alameda Professor Hernâni Monteiro, 4200-319 Porto, Portugal; 3 CINTESIS (Centro de Investigação em Tecnologias da Saúde e Sistemas de Informação em Saúde), Serviço de Bioestatística e Informática Médica, Faculdade de Medicina da Universidade do Porto, Alameda Professor Hernâni Monteiro. 4200-319 Porto, Portugal

## Abstract

**Background:**

Acute kidney injury (AKI) has been hard to assess due to the lack of standard definitions. Recently, the Risk, Injury, Failure, Loss and End-Stage Kidney (RIFLE) classification has been proposed to classify AKI in a number of clinical settings. This study aims to estimate the frequency and levels of severity of AKI and to study its association with patient mortality and length of stay (LOS) in a cohort of trauma patients needing intensive care.

**Methods:**

Between August 2001 and September 2007, 436 trauma patients consecutively admitted to a general intensive care unit (ICU), were assessed using the RIFLE criteria. Demographic data, characteristics of injury, and severity of trauma variables were also collected.

**Results:**

Half of all ICU trauma admissions had AKI, which corresponded to the group of patients with a significantly higher severity of trauma. Among patients with AKI, RIFLE class R (Risk) comprised 47%, while I (Injury) and F (Failure) were, 36% and 17%, respectively. None of these patients required renal replacement therapy. No significant differences were found among these three AKI classes in relation to patient's age, gender, type and mechanism of injury, severity of trauma or mortality. Nevertheless, increasing severity of acute renal injury was associated with a longer ICU stay.

**Conclusions:**

AKI is a common feature among trauma patients requiring intensive care. Although the development of AKI is associated with an increased LOS it does not appear to influence patient mortality.

## Introduction

Acute Kidney Injury (AKI) affects 5 to 7% of all hospitalized patients. In the ICU population, this syndrome is common with an incidence of 1 to 25%, depending on the criteria used for definition, and is associated with mortality rates of 50 to 70% [1-6]. For many decades, diverse definitions for AKI have been used, which explains the difficulty in understanding the wide inter-study variations. AKI is a complex disorder with multiple etiologies, different clinical manifestations, and outcomes ranging from minimal elevation in serum creatinine to anuric renal failure.

In response to the need for a common meaning for AKI, because AKI has been, over the last few decades the focus of extensive clinical research efforts, the Acute Dialysis Quality Initiative Group, a panel of international experts in nephrology and critical care medicine, developed and published a set of consensus criteria for a uniform definition and classification of AKI [7] (table 1 shows the RIFLE classification). These criteria, which make up the acronym 'RIFLE', classify renal dysfunction according to the degree of impairment present: there are three grades of severity - risk (R), injury (I), and failure (F), and two outcome classes - sustained loss (L) of kidney function and end-stage kidney disease (E). RIFLE criteria, which have the advantage of providing diagnostic definitions for a stage when kidney injury can still be prevented (R), have been tested in clinical practice and seem to be at least congruent with the outcome of a patient with AKI [8-10]. This system has several advantages. It appears sensitive to the early changes in kidney function, allows monitoring of progression of AKI and could function as a robust instrument to discriminate clinical relevant outcomes. The RIFLE classification has been evaluated and validated in numerous clinical studies enrolling critically ill patients namely post-operative patients and burned patients, and found to be a valid tool for the precocity of the diagnosis and staging of AKI, having predictive ability for mortality [11-16].

**Table 1:** Risk, Injury, Failure, Loss and End-stage Kidney (RIFLE) classification [7].

Class	Glomerular filtration rate criteria	Urine output criteria
**Risk**	Increased SCreat ×1.5 or GFR decrease >25%	<0.5 ml/kg/hour × 6 hours
		
**Injury**	Increased SCreat ×2 or GFR decrease >50%	<0.5 ml/kg/hour × 12 hours
		
**Failure**	Increased SCreat ×3 or GFR decrease >75% or SCreat ≥ 4 mg/dl	<0.3 ml/kg/hour × 24 hours, or anuria × 12 hours
		
**Loss**	Persistent acute renal failure = complete loss of kidney function > 4 weeks	
		
**End-stage kidney disease**	End-stage kidney disease > 3 months	

A few studies in trauma patients have shown that the incidence of renal failure varies from less than 0.1% to 18%, with an associated mortality ranging from 7 to 83% [12,13,17-19]. In particular, the study by Bagshaw and a study by Yuan were able to show the application of the RIFLE criteria to characterize AKI in a population of patients with trauma [12,19].

Keeping in mind the relevance of this issue and the limited data available in the literature, we aimed to characterize AKI using the RIFLE classification and relate it to ICU length of stay (LOS), hospital LOS, and mortality in a cohort of severe trauma patients that needed Intensive Care. Preliminary results of this study were published elsewhere [20].

## Materials and methods

We studied all trauma patients admitted to the ICU between August 2001 and September 2007 at Hospital de Santo António. This university hospital is a level 1 trauma centre in the city of Porto in northern Portugal, with about 1800 trauma patients per year [21].

Epidemiology and severity data including age, gender, mechanism of injury, injury severity score (ISS), revised trauma score (RTS), Trauma and Injury Severity Score (TRISS) [22], and length of stay were obtained from the prospective trauma registry. TRISS methodology is one of the most used severity methodologies. It uses anatomic severity (ISS) and physiological severity (RTS), age and type of trauma to arrive to a probability of survival. Clinical charts were reviewed for urine output, daily serum creatinine, intracranial hypertension and Simplified Acute Physiology Score (SAPS II). Patients with chronic kidney disease and a second admission were excluded. Chronic kidney disease was defined using the definition of the National Kidney Foundation [23]. Intracranial hypertension was defined as persistent intracranial pressure above 20 mmHg. Renal trauma was defined as direct trauma to the kidney resulting from the accident.

Patients were classified into classes R (Risk), I (Injury) and F (Failure), according to the highest RIFLE class reached during their ICU stay. The RIFLE class was determined according to the worst degree of either glomerular filtration rate (GFR) criteria (according to the creatinine values and never used the GFR per se) or urine output criteria. For patients without serum creatinine baseline historical data, we determined a baseline serum creatinine level using the Modification of Diet in Renal Disease equation (MDRD) [24]. When baseline serum creatinine is unknown, current recommendations allow you to estimate this value using the MDRD equation, assuming a glomerular filtration ratio of 75 ml/min/1.73 m^2^. Recently, Bagshaw and collaborators validated the use of this equation to assess RIFLE criteria [25].

We measured outcomes as the use of renal replacement therapy, length of ICU and hospital stay, and mortality. We divided mortality into ICU mortality, if it occurred during ICU stay and Hospital mortality if it occurred during the rest of Hospital stay. If mortality occurred after hospital discharge it was not considered. Moreover we divided mortality into early (2 or less days) and late (more than 2 days).

Continuous variables were expressed as means ± standard deviations for normal distributed variables and medians and inter-quartile range (IQR) otherwise. The categorical variables were expressed as absolute and relative frequencies. Pearson Chi Square was used to analyze categorical data. ANOVA and T test were used for variables with normal distributions, and Mann Whitney or Kruskall Wallis for other data. A P-value < 0.05 was considered statistical significant. Analysis was performed with the statistical software package SPSS 15.0 for Windows.

## Results

In total, 436 trauma patients admitted in ICU were studied. Patients characteristics, outcomes, and comparison between AKI and no AKI groups are summarized in table 2 and 3. All the patients were mechanically ventilated. Eighty percent of patients were male, with a median age of 37 years (IQR 23-55). The majority had blunt trauma (95%) caused by road traffic accidents (67%). Mean ISS and RTS was 27.3 (SD = 11.4) and 5.7 (SD = 1.4), respectively. Renal trauma had an incidence of 2.5% in our cohort (11 patients), with a similar distribution in the AKI and non AKI groups.

**Table 2:** Population characteristics.

		All trauma		
			
	Total (n = 436)	No AKI (n = 219, 50%)	AKI (N = 217, 50%)	p
**Baseline characteristics**				
**Gender**, n (%)				
Male	350 (80)	170 (78)	180 (83)	0.162
Female	86 (20)	49 (22)	37 (17)	
**Age**, median (IQR)	37 (23-55)	37 (22-52)	37 (24-55)	0.814
**ISS**, mean (SD)	27.3 (11.4)	26.2 (10.9)	28.4 (11.8)	**0.045**
**TRISS**, mean (SD)	71.2 (27.1)	70.1 (27.2)	68.1 (28.00)	0.414
**SAPS II**, median (IQR)	36 (26-45)	34 (25-45)	38 (28-46)	0.288
**Intracranial hypertension, n (%)**	254 (58)	167 (76)	87 (40)	**<0.001**
**Trauma, n (%)**				
Head	410 (94)	205 (94)	205 (95)	0.704
Thorax	216 (50)	107 (49)	109 (50)	0.775
Abdomen	54 (12)	22 (10)	32 (15)	0.136
Pelvis and limbs	202 (46)	94 (43)	108 (50)	0.152
Spinal	17 (4)	6 (3)	11 (5)	0.209
**Renal trauma, n(%)**	11 (2.5)	5 (2.3)	6 (2.8)	0.748
				
**Outcomes**				
				
**ICU LOS, median (IQR)**	7 (3-13)	5 (2-11)	9 (5-16)	**<0.001**
**Hospital LOS, median (IQR)**	13 (5-24)	10 (3-19)	16 (9-29)	**<0.001**
**ICU mortality, n (%)**	97 (22)	61 (28)	36 (17)	**0.005**
**Hospital Mortality (n%)**				
**Overall**	129 (30)	82 (37)	47 (22)	**<0.001**
**Early**	57 (13)	45 (21)	12 (6)	**<0.001**
**Late**	72 (17)	37 (22)	35 (18)	0.315

**Table 3:** AKI patient's characteristics

	Only AKI patients			
		
	Risk (n = 102, 24%)	Injury (n = 78, 18%)	Failure (n = 37, 8%)	p
**Baseline characteristics**				
**Gender**, n (%)				
Male	88 (86)	65 (83)	27 (73)	0.182
Female	14 (14)	13 (17)	10 (27)	
**Age**, median (IQR)	40 (24-55)	36 (22-57)	35 (28-53)	0.626
**ISS**, mean (SD)	27.7 (10.96)	29.0 (13.1)	28.9 (11.7)	0.736
**TRISS**, mean (SD)	69.2 (27.5)	67.00 (28.9)	66.6 (28.4)	0.831
**SAPS II**, median (IQR)	38 (15-75)	38 (31-48)	36 (26-42)	0.299
**Trauma, n (%)**				
Head	98 (96)	75 (95)	32 (87)	-
Thorax	49 (48)	36 (46)	24 (65)	0.143
Abdomen	18 (18)	8 (10)	6 (16)	0.368
Pelvis and limbs	49 (48)	36 (46)	23 (62)	0.246
Spinal	5 (5)	6 (8)	0(0)	-
**Renal trauma, n(%)**	5 (4.9)	0 (0)	1 (2.7)	**-**
				
**Outcomes**				
				
**ICU LOS, median (IQR)**	8 (5-12)	9 (7-17)	13 (7-19)	**<0.044**
**Hospital LOS, median (IQR)**	15 (7-30)	17 (9-24)	18 (9-33)	<0.696
**ICU mortality, n (%)**	15 (15)	14 (18)	7 (19)	0.775
**Hospital Mortality (n%)**				
**Overall**	24 (23)	15 (19)	8 (22)	0.786
**Early**	7 (7)	2 (3)	3 (8)	-
**Late**	17 (19)	13 (18)	5 (15)	0.885

The highest RIFLE class was obtained using serum creatinine in 98.6% of patients and using urine output in 1.4% of patients (3 patients only). In 76.1% of the patients the baseline serum creatinine was calculated using the MDRD equation because a record with previous baseline levels was not present for most of the patients. Concerning urinary output all patients except the 3 mentioned had more than 0.5 ml/Kg/h of diuresis. In all other patients what gave the RIFLE class of Risk, Injury or Failure was the increase from the basal level of creatinine to the maximum level of creatinine achieved during the entire length in ICU according to the criteria defined in table 1[7].

AKI occurred in 217 patients (50%) but only 8% developed class F. No differences in age, gender, type of injury, mechanism of injury, TRISS, SAPS II, incidence of different body regions involved or RTS were found between patients with and without AKI. The severity of trauma, assessed by ISS, was higher in the AKI group (28.4 ± 11.8 *vs. *26.21 ± 10.9, p = 0.045). In the subgroup of patients with AKI, 47% had a maximum RIFLE class of Risk, 36% had Injury, and 17% had Failure.

In terms of outcomes, none of the patients in our study required renal replacement therapy during ICU or hospital stay, and no patients reached the RIFLE outcome classes L or E. All patients that survived returned to normal levels of creatinine and diuresis. Increasing severity of AKI was associated with a significant increase in ICU length of stay (p = 0.044). Length of hospital stay also tended to increase with severity of AKI, but the differences had no statistical significance. We were not able to relate an increase in mortality to the severity of AKI. Overall trauma patient mortality was 30% and was significantly higher for patients without AKI. Regarding late mortality, no differences were found between the AKI and No AKI groups (18% versus 22%, p = 0.315). When stratified by RIFLE category the crude mortality was 23% for Risk, 19% for Injury, and 22% for Failure.

To better understand mortality distribution we divided the mortality into early (less than two days) and late categories. We found a significantly higher proportion of mortalities in the first two days in the group of patients without AKI (79% of early deaths are in the No AKI group). We also found a significantly higher incidence of intracranial hypertension and a higher proportion of mortality due to intracranial hypertension in the No AKI group of patients.

## Discussion

Our main finding was that AKI (defined using RIFLE criteria) was common in ICU trauma patients. Recently, Bagshaw et al. suggested that trauma admissions to the ICU are frequently complicated by early AKI, with an incidence of about 18% [12]. Despite their use of the RIFLE classification, comparison of the results of that study and this one is difficult. That study only looked at early AKI while this study looked at the full range of AKI and found an even higher AKI incidence (50%). RIFLE criteria have recently been used to define AKI in a variety of ICU patients, and in accordance with other studies, we also found that RIFLE allows for the identification and classification of a significant proportion of critically injured patients as having some degree of AKI [8,10,12,13]. We found that the development of AKI was related to the severity of illness, in the case of trauma assessed by the ISS, but not to age, gender, type of trauma or mechanism of injury. A recent paper by Yuan finds an incidence of AKI defined also by RIFLE in only 10.7% of all road traffic accidents. Yuan describes a cohort of trauma patients admitted only after road traffic accident and that had also minor traumas [19]. We describe a cohort of severely injured trauma patients admitted to the ICU. That helps explain the differences in incidence of AKI between our study and the two studies that also use RIFLE criteria in trauma patients.

A second important finding was that the development of AKI, defined by the RIFLE criteria, had consequences in terms of outcome, namely an increase in ICU and hospital LOS, but did not result in the need for renal replacement therapies (RRT) in any of our patients. Besides we found AKI in 50% of patients, most of the patients had classes Risk and Injury and only 8% had Failure. Most of the studies that address AKI in trauma suggest that AKI is rare and that the use of renal replacement therapy is even rarer and usually related to the development of severe sepsis [18]. In this paper Brown et al. find a need for RRT in only 0.2% of trauma patients. One result that is probably difficult to generalize to other ICU or country is the absence of renal replacement therapy. Indication and timing for RRT varies in different countries and institutions and our result probably reflect also local policies. The small number of patients (37) that were classified as Failure according to RIFLE criteria might also have limited the study of RRT outcome.

Finally we did not find a relation between AKI (and the R, I and F RIFLE categories) and mortality. Early mortality for non AKI patients was dependent on the severity of the head injury related to the development of intracranial hypertension. That was not a surprise as we know that the main causes of death in trauma are bleeding and head trauma. We were however surprised by the absence of relationship between later mortality and AKI. We can hypothesize that the reasons might be related to an improved pre-hospital and emergency room care or less co-morbidity in the population studied or less sepsis in the ICU population compared to other studies. However we do not have data in the present study to confirm those hypotheses.

The retrospective nature of this study is a limitation, especially since we did not have any pre-ICU data in most of the patients, such as previous creatinine values. In addition, this study was performed at a single level I trauma centre and a single ICU and the case mix might affect the detection of outcomes of interest and the generalization of the conclusion. However concerning the capture of outcome of interest - AKI - we consider that this cohort is highly representative as it is constituted by the most severe patients expected to progress to AKI. Studies have suggested that AKI in trauma develops late and as a complication of multiple organ dysfunction syndromes [18,26]. We again consider that the ICU setting would be most appropriate to capture AKI. Possibly we could have overestimates the incidence of AKI in our cohort compared to a cohort of less severe patients. The incidence of AKI could have been lower in a different ICU with different patient severity. However considering that our case mix of very severe trauma patients admitted to ICU is the most appropriate to study AKI we would not expect to see a different relation between AKI and mortality in a less severe cohort of patients.

## Conclusions and further research

In a population of severe trauma patients admitted to the ICU, AKI was frequent and associated with an increase in ICU and hospital stay but not with mortality. Further research, with a prospective design addressing etiology and time to AKI is needed to help in the discussion of the relationship between AKI and mortality in severe trauma patients.

## Competing interests

The authors declare that they have no competing interests.

## Authors' contributions

EG and RA carried out the design of the study, acquisition of data, analysis and interpretation of data and drafted the manuscript. CD participated in the design of the study and performed the statistical analysis. RA and ACP participated in the design of the study and helped to draft the manuscript. All authors read and approved the final manuscript.
